# Supplementary material for: A practical tool for maximal information coefficient analysis
Source: Gigascience. 2018 Apr 2;7(4):1–8. doi: 10.1093/gigascience/giy032 (PMC5893960; doi:10.1093/gigascience/giy032)
Supplement: GIGA-D-17-00300_Revision_1.pdf [file giy032_giga-d-17-00300_revision_1.pdf]

GigaScience

A practical tool for Maximal Information Coefficient analysis

--Manuscript Draft--

**Manuscript Number:**	GIGA-D-17-00300R1
**Full Title:**	A practical tool for Maximal Information Coefficient analysis
**Article Type:**	Technical Note
**Funding Information:**	Autonomous Province of Trento (Accordo di Programma) — Mr Davide Albanese
**Abstract:**	Background.<br>The ability of finding complex associations in large omics datasets, assessing their significance, and prioritizing them according to their strength can be of great help in the data exploration phase. Mutual Information based measures of association are particularly promising, in particular after the recent introduction of the TICe and MICe estimators, which combine computational efficiency with superior bias/variance properties. An open-source software implementation of these two measures providing a comprehensive procedure to test their significance would be extremely useful.<br><br>Findings.<br>In this paper we present MICtools, a comprehensive and effective pipeline which combines TICe and MICe into a multi-step procedure that allows the identification of relationships of various degrees of complexity. MICtools calculates their strength assessing statistical significance using a permutation-based strategy. The performances of the proposed approach are assessed by an extensive investigation in synthetic datasets and an example of a potential application on a metagenomic dataset is also illustrated.<br><br>Conclusions<br>We show that MICtools, combining TICe and MICe, is able to highlight associations that would not be captured by conventional strategies. MICtools is implemented in Python, and is available for download at https://github.com/minepy/mictools.
**Corresponding Author:**	Davide Albanese<br><br>ITALY
**Corresponding Author Secondary Information:**	
**Corresponding Author's Institution:**	
**Corresponding Author's Secondary Institution:**	
**First Author:**	Davide Albanese
**First Author Secondary Information:**	
**Order of Authors:**	Davide Albanese
	Samantha Riccadonna
	Claudio Donati
	Pietro Franceschi
**Order of Authors Secondary Information:**	
**Response to Reviewers:**	Dear Editor,<br>on behalf of all authors, I’m pleased to submit the revised manuscript ”A practical tool for Maximal Information Coefficient analysis” by Albanese et al., to be considered for publication in Gigascience as Technical Note.<br><br>We are grateful to the referees for their time and efforts: their comments has been extremely useful in improving the overall quality of the manuscript and we incorporated

Powered by Editorial Manager® and ProduXion Manager® from Aries Systems Corporation

all their suggestion in the revised text. Hereafter, the answers to the referees comments are highlighted by the string ">>>".

The authors declare no conflict of interest and have approved the manuscript for submission. I hereby confirm that the present work has not been published and submitted for publication elsewhere.

Editor's comments

Please register any new software application in the SciCrunch.org database to receive a RRID (Research Resource Identification Initiative ID) number, and include this in your manuscript. This will facilitate tracking, reproducibility and re-use of your tool.

>>> The software tool has been registered in SciCrunch as SCR_016121. This information has been included in the manuscript.

A third referee declined to review as they had very strong views against maximal information coefficient (MIC), feeling it was essentially a messed-up estimator of mutual information, and that any research based on it was flawed. Consulting with the other referees they disagreed with this, but it does seem there are strong views on this technique. It is probably not possible to satisfy both sides of this debate, but if there are any ways to acknowledge this in the discussion that could potentially be useful.

>>> We are aware the the introduction of MIC has been triggering a lively discussion in the scientific community. In order to acknowledge that we added a short sentence in the introduction.

Reviewer #1

In this paper the authors describe and analyse a series of tools to find complex associations in large omics data sets. At the core of these tools lies the measure of association Maximal Information Coefficient (MIC) which recently received a lot of interest in data mining community. Other than presenting the first publicly available implementation of MIC to date, the authors make available the code for a complete pipeline to identify statistically significant associations between the features in a data set. This involves:
- Computing the Total Information Coefficient (TIC) for each pair of features
- Computing their p-value using a permutation test with Monte Carlo simulations
- Select the significant pairs using statistical correction for multiple hypotheses
- Rank the statistically significant associations according to MIC

Moreover, the authors analyse the results of their pipeline on synthetic and real data sets. I commend the authors for providing the community with a well-tested implementation of MIC (and its more recent version MIC_e) in various programming languages including C, Matlab, and Python. I also really appreciate publishing a full pipeline to identify associations between features written in Python, which is probably the most popular language in the data science community. Moreover, the paper is well written and the analyses about the effectivity of these tools are convincing. The paper should be accepted for publication in the GigaScience journal. There has been so much discussion about the merit of MIC in the past years since its publication in 2011. I am honestly impressed by MIC's authors efforts to shed light on the theoretical and empirical properties of MIC. Their effort recently found venue in prestigious journals such as the Proceedings of the National Academics of Science (PNAS) in 2014, the Journal of Machine Learning Research (JMLR) in 2016, and the Annals Of Applied Statistics (AOAS) in 2017. The main criticism about MIC has been its similarity to one of the many estimators of mutual information. Even though MIC exploits mutual information, MIC has been shown to not be the same as estimating mutual information [Measuring dependence powerfully and equitably by Reshef et al. in JMLR 2016]. Nonetheless, what strikes me the most is that: in many empirical studies no estimator of mutual information has the same performance of MIC in terms of equitability. Being equitability a very intuitive property, I do understand why researchers and data mining practitioners value MIC. I have only one concern about the methodology of screening

associations with TIC and ranking only the selected ones with MIC. Possibly if we are interested just in equitability, MIC should be the only association measure to be employed in the analysis. However, given than TIC shows to have more power the MIC [An Empirical Study of the Maximal and Total Information Coefficients and Leading Measures of Dependence by Reshef et al. in AOAS 2017], I guess that the associations that MIC would deem as significant would be a subset of the significant associations for TIC.

>>> We thank the reviewer for his comment and exactly for the reasons he mention we decided to rely on a two step procedure to look for an optimal trade-off between speed/power and equitability

Minor comments:
It would be great to describe the Storey's method to control the FDR in the paper to make it self-contained; It would be also great to briefly describe the procedure to control the FWER;

>>>The section describing how multiple testing correction was performed has been extended including:
>>> - a definition of FWER and FDR
>>> - an explicit description of the key idea behind Storey’s q-value

A table describing the difference between the data sets SD1 and SD2 would be informative. Possibly a line describing the Madelon semi-synthetic data sets would be useful too

>>> A description of the characteristics of the two synthetic dataset gas been included in the main text as Table 2

The authors discuss a great insight on MIC when they say that: "associations between informative/redundant and redundant/redundant variables were significant also for a lower number of samples". It would be nice to have a visual example about these type of associations;

>>> Figure 4 was modified to include four visual examples of the associations found in the Madelon dataset. In particular two examples of IR and RR associations for 50 and 500 samples are included in the panel (d) of the revised figure.

Figure 4 b. I guess discussing a decreasing FN is the same as discussing increasing power. Changing the FN plot in a power plot would make the paper more coherent: eg as in Figure 2 a;

>>> The figure was corrected following the Referee’s suggestion

"coniugate" in the abstract -> conjugate. Maybe better to reformulate this sentence as it is not very clear;

>>> The corresponding sentence has been rephrased also according to the comments of Reviewer #2

Reviewer #2

This manuscript introduces an open-source implementation of two measures of dependence, MICe and TICe, which together provide a combination of both statistical power and equitability for identifying associations in large data sets. The implementation provided by the authors is a valuable contribution to the community that allows for the easy computation of these measures of dependence, and I'd recommend its acceptance after the authors make the minor edits listed below.

Minor Comments
A few minor comments that the authors should be made aware of (but that I didn't want

Powered by Editorial Manager® and ProduXion Manager® from Aries Systems Corporation

	to be public given how minor they are):<br><br>There are a few small type-o's to correct (e.g. coniugate on Pg. 1, line 31; expenses on pg. 2, line 15).<br><br>>>> Corrected<br><br>I would suggest the authors soften the language around the fact that "an implementation of these two measures and of a statistical procedure to test the significance of each association is still missing." The authors who developed MICe and TICe are simply waiting to post their implementation of MICe and TICe at www.exploredata.net along with the official publication of the most recent paper analyzing these measures in the Annals of Applied Statistics (https://www.e-publications.org/ims/submission/AOAS/user/submissionFile/29563?confirm=583655c8) . That said, the implementation in this manuscript submitted to GigaScience is still a valuable contribution as it is open-source (the implementation AOAS will post is not) and provides a more comprehensive procedure to test for significance.<br><br>>>> The text has been corrected following the referee's advice<br><br>On Pg. 1, line 31, "which coniugate computational efficiency with good bias/variance properties", isn't quite accurate. I'd change this to "which combine computational efficiency with superior bias/variance properties".<br><br>>>> Changed<br><br>On Pg. 2, line 5, "has been shown to satisfy the equitability requirement" should be changed to "has been shown to have good equitability" to reflect the fact that equitability is not a binary property, but a continuous one that a measure of dependence can have more or less of.<br><br>>>> Changed<br><br>On Pg. 2, line 6 - MIC doesn't actually suffer from lack of power, and this fact has been corrected in the literature, so I would recommend using softer language. It was shown in ref. 12 that was cited by the authors that the original perceived bad power of MIC was due to incorrect parameter settings by those who drew that conclusion. When used with appropriate parameters for independence testing, MIC has decent, but not state-of-the-art, power. What is accurate, however, is that MICe and TICe *improved* upon the power of MIC, and that TICe has state-of-the-art power.<br><br>>>> "suffers of lack of power" has been changed with "does not have state-of-the-art power"<br><br>On Pg. 2, second column, line 23, regarding the sentence beginning with "With regards to the number of permutations…" (and elsewhere): the number of permutations necessary to perform for any given analysis scales with the number of tests one must correct for (i.e. the number of variable pairs for which a measure of dependence was computed), as the FDR accuracy is inversely proportional to the number of permutations used to compute it, so I'd be careful about saying that a specific number is generally enough for data of any dimensionality.<br><br>>>> The text of the paper was changed according to the referee's comments. In particular we highlighted the fact that the the number of permutations is a parameter that can be adjusted by the user on the bases of the dataset characteristics.
**Additional Information:**	
**Question**	**Response**
Are you submitting this manuscript to a special series or article collection?	No

Powered by Editorial Manager® and ProduXion Manager® from Aries Systems Corporation

**Experimental design and statistics**<br><br>Full details of the experimental design and statistical methods used should be given in the Methods section, as detailed in our Minimum Standards Reporting Checklist. Information essential to interpreting the data presented should be made available in the figure legends.<br><br>Have you included all the information requested in your manuscript?	Yes
**Resources**<br><br>A description of all resources used, including antibodies, cell lines, animals and software tools, with enough information to allow them to be uniquely identified, should be included in the Methods section. Authors are strongly encouraged to cite Research Resource Identifiers (RRIDs) for antibodies, model organisms and tools, where possible.<br><br>Have you included the information requested as detailed in our Minimum Standards Reporting Checklist?	Yes
**Availability of data and materials**<br><br>All datasets and code on which the conclusions of the paper rely must be either included in your submission or deposited in publicly available repositories (where available and ethically appropriate), referencing such data using a unique identifier in the references and in the "Availability of Data and Materials" section of your manuscript.<br><br>Have you have met the above requirement as detailed in our Minimum Standards Reporting Checklist?	Yes

Powered by Editorial Manager® and ProduXion Manager® from Aries Systems Corporation

Manuscript Click here to download Manuscript main.tex

OXFORD (GIGA)^n SCIENCE

GigaScience, 2017, 1–8

doi: xx.xxxx/xxxx
Manuscript in Preparation
Paper

PAPER

A practical tool for Maximal Information Coefficient analysis

Davide Albanese[1], Samantha Riccadonna[1], Claudio Donati[1] and Pietro Franceschi[1,*]

[1] Computational Biology Unit, Research and Innovation Centre, Fondazione Edmund Mach, via E.Mach 1, 38010 S. Michele all'Adige (TN), Italy

*pietro.franceschi@fmach.it

Abstract

Background. The ability of finding complex associations in large omics datasets, assessing their significance, and prioritizing them according to their strength can be of great help in the data exploration phase. Mutual Information based measures of association are particularly promising, in particular after the recent introduction of the TIC_e and MIC_e estimators, which combine computational efficiency with superior bias/variance properties. An open-source software implementation of these two measures providing a comprehensive procedure to test their significance would be extremely useful. **Findings**. In this paper we present MICtools, a comprehensive and effective pipeline which combines TIC_e and MIC_e into a multi-step procedure that allows the identification of relationships of various degrees of complexity. MICtools calculates their strength assessing statistical significance using a permutation-based strategy. The performances of the proposed approach are assessed by an extensive investigation in synthetic datasets and an example of a potential application on a metagenomic dataset is also illustrated. **Conclusions**. We show that MICtools, combining TIC_e and MIC_e, is able to highlight associations that would not be captured by conventional strategies. MICtools is implemented in Python, and is available for download at https://github.com/minepy/mictools.

Key words: Maximal information coefficient; MIC; TIC; equitability; multiple testing; permutation test; power of statistical significance; false discovery rate; FDR

Introduction

With the growing popularity of high throughput quantitative technologies it is now common to characterize living systems by measuring thousands of variables over a wide range of conditions. In these large datasets, the number of potential associations between variables is enormous. Computational and statistical methods should be able to highlight the significant ones (striking a balance between flexibility and statistical robustness), and to prioritize the more relevant for downstream analysis. Traditionally, the presence of a potential relationship between two variables X and Y is assessed on the basis of a certain measure of association, that is often able to reveal specific types of relationships, but is blind to others. Then, once the measure is computed, its significance is tested against the null hypothesis of no association. For linear associations, the Pearson correlation coefficient is the natural choice, while the Spearman's rank coefficient represents a more flexible alternative for general monotonic relationships. In the exploratory analysis of datasets produced by modern -omics technologies this conventional approach show its limits, because a huge number of potential associations needs to be screened without any *a priori* information on their form. In these cases, it would be desirable to use a measure of dependence that ranks the relationships according to their strength, regardless of the type of association. A measure with this property has been defined *equitable* [1] and a consistent mathematical framework for the definition of equitability has been proposed [2, 3, 4, 5, 6]. The second challenge faced in the unsupervised screening of large

Compiled on: March 8, 2018.
Draft manuscript prepared by the author.

datasets is that the number of associations to be tested is usually huge and the statistical assessment of significance has to face well known multiplicity issues [7, 8].

Recently, a family of measures based on the concept of mutual information has been proposed, and one of the most popular (and debated) members of this family, the Maximal Information Coefficient (MIC), has been shown to have good equitability [1]. Unfortunately, MIC does not have state-of-the-art power [9, 10], and its heuristic estimator, APPROX-MIC, is computationally demanding [5]. These two drawbacks have severely hampered the application of MIC to large datasets. In order to overcome these limitations, two new MIC-based measures, the MIC_e— a consistent estimator of the MIC population value (MIC_*) — and the related TIC_e (total information coefficient) statistics have been proposed [5]. Both quantities can be calculated more efficiently than APPROX-MIC and have better bias/variance properties [5]. In particular, TIC_e is characterized by high power, which has been obtained at the cost of equitability, while MIC_e performs better on this side, showing reduced performances in terms of power. These two MIC-based measures, then, compensate each other and their combination is extremely promising as data exploration tool. In particular, a two step procedure can be applied, where TIC_e is used to perform efficiently a high throughput screening of all the possible pairwise relationships and assess their significance, while MIC_e is used to rank the subset of significant associations in terms of strength [5]. Despite the potential of this approach, an efficient software implementation of these two measures and of a statistical procedure to test the significance of each association controlling multiplicity issues is still lacking.

Here we present MICtools, an open-source and easy-to-use software providing:

- an efficient implementation of TIC_e and MIC_e estimators[11];
- a permutation-based strategy for estimating TIC_e empirical p values;
- several methods for multiple testing correction, including the Storey's q value to control the false discovery rate (FDR);
- the MIC_e estimates for each association called significant.

Methods

MICtools implements a multi-step procedure to identify relevant associations amongst a large number of variables, assess their statistical significance and rank them according to the strength of the relationship. Starting from M variable pairs x_i and y_i measured in n samples, the procedure can be broken into 4 steps (Figure 1):

(i) estimating the empirical TIC_e null distribution by permutations;
(ii) computing TIC_e statistics and its empirical p values for each variable pairs;
(iii) applying a multiple testing correction strategy in order to control the family-wise error rate (FWER) or the FDR [12];
(iv) using MIC_e to estimate the strength of the relationships called significant.

The pipeline can be run as a sequence of subcommands implemented into the main command `mictools` (Figure 1).

The empirical TIC_e null distribution

Since TIC_e depends only on the rank-order of the vectors x_i and y_i [1], the empirical null distribution can be estimated, for a given sample size and set of parameters, by performing R permutations of the elements of the vectors y_i and by calculating the set of null TIC_e statistics $t_1^0, \ldots, t_R^0$. Two parameters control the estimation of the null distribution of TIC_e: the parameter B controlling the maximal-allowed grid resolution and the number of permutations R. In the current implementation, B was set to the default value 9, which guarantees good performances in terms of statistical power against independence in most situations [10]. However, different values of B can be chosen: for example, $B = 4$ for less complex alternative hypothesis, $B = 12$ for more complex associations [10]. With regards to the number of permutations, instead, the results obtained on the synthetic datasets (see Additional File 2, Figures A2 and A3 and Additional File 1, Table A2) empirically indicate that 200,000 permutations represent a reasonable choice for the dataset SD1 (see the Methods section).

Computing the TIC_e and its associated empirical p values for each variable pair

The total information coefficient is computed for each (non permuted) variable pair, obtaining a set of TIC_e values t_i (with $i = \{1, \ldots, M\}$). For each t_i, the p value p_i is estimated as the fraction of values of the empirical null distribution that exceeds t_i [13]:

$$p_i = \frac{1 + \#\{r : t_r^0 \geq t_i, \quad r = 1, \ldots, R\}}{1 + R}$$

Multiple testing correction

Considering the large number of tests of independence performed, it is necessary to correct the p values for multiplicity. In general, this can be done either by controlling the Family Wise Error Rate (FWER) or the False Discovery Rate (FDR). The first approach aims at controlling the probability of making at least one Type I error in the set of tests and this is done by decreasing the significance threshold of each individual test (as in Bonferroni correction). In the case of FDR, instead, the presence of false positives is accepted and what is controlled is their fraction among the associations called significant. This is done by estimating the distribution of the p values under the hypothesis of independence and comparing it with the observed one. MICtools implements several state-of-the-art strategies to accomplish this task. For all the examples presented here we have used the Storey's method for estimating the q values to control the FDR [7]. Assuming a uniform distribution for the null p values, the fraction of associations for which the null is true (π_0) is estimated directly from the shape of distribution at high p values. π_0 is then used to calculate the q value for the ith association as the minimum FDR which can be obtained varying the significance threshold (h):

$$q(p_i) = \min_{h \geq p_i} \mathrm{FDR}(h) = \min_{h \geq p_i} \frac{\pi_0 M h}{\#\{p_i \leq h\}}.$$

Briefly, setting a q-value cut-off to 0.05, we accept a FDR of at most 5%. To check the method assumptions MICtools provides the empirical distribution of p values as diagnostic plot.

Computing the MICe on the significant relationships

Finally, the strength of the associations that pass the significance threshold is estimated using the MIC_e estimator. In this case, we define the the B parameter as a function of the number of samples n, $B(n) = n^{\alpha}$ [1]. The default values are optimized

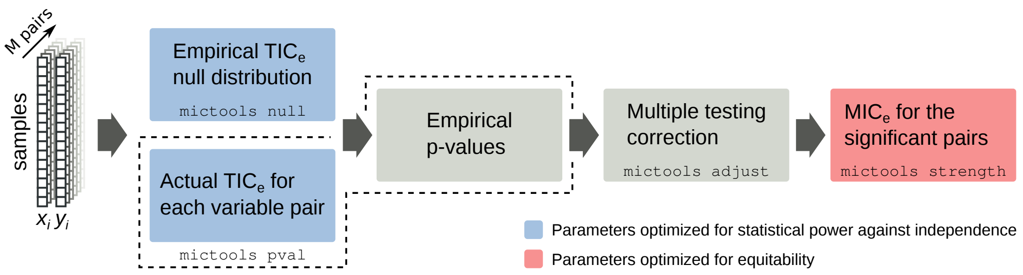

Figure 1. The MICtools pipeline. Each step is implemented as a subcommand of the `mictools` main command. `mictools null` estimates the empirical TIC$_e$ null distribution of the M variable pairs (x_i, y_i). `mictools pval` computes the TIC$_e$ values and estimates their p values (boxes withing the dashed line). The multiple testing correction is performed by `mictools adjust`. Finally, `mictools strength` estimates the MIC$_e$ value for the subset of significant relationships. The color of the boxes highlights the criterion used for parameter optimization.

Table 1. The default values of the α parameter vary according to the number of samples.

Number of samples n	α parameter
$n < 25$	0.85
$25 \leq n < 50$	0.80
$50 \leq n < 250$	0.75
$250 \leq n < 500$	0.70
$500 \leq n < 1,000$	0.65
$1,000 \leq n < 2,500$	0.60
$2,500 \leq n < 5,000$	0.55
$5,000 \leq n < 10,000$	0.50
$10,000 \leq n < 40,000$	0.45
$n > 40,000$	0.40

for equitability [6] and summarized in Table 1.

Findings

Two synthetic datasets (SD1 and SD2) were created in order to assess (i) the statistical power (or recall, i.e. the fraction of non-independent relationships that were recovered at a given significance level) and (ii) the ability to control the FDR. The analyses were performed varying the number of samples (SD1) and the effect chance [14], i.e. the percentage of non independent variable pairs (SD2). Both datasets contain a set of independent variables and a fixed number of variable pairs X and Y related by associations in the form $Y = f(X) + \eta$, where $f(X)$ is a function and η is a noise term. To characterize the performances of MICtools in presence of associations that could not be described by a function, a series of Madelon datasets [15, 16] was also analyzed. The main characteristics of the three synthetic datasets are summarized in Table 2. Finally, the proposed pipeline was applied to the analysis of an environmental/metagenomic dataset which has been recently made available within the Tara project, a global-scale characterization of plankton using high throughput metagenomic sequencing [17].

Table 2. Characteristics of the three synthetic datasets analyzed in this work.

	SD1	SD2	Madelon
N. associations	60,000	60,000	19,900
Effect chance (%)	1	1, 2, 5, 10, 20, 50	1
N. samples	25, 50, 100, 250, 1,000	100	50, 250, 1,000, 2,500, 5,000
N. replicates	20	20	1
Total n. replicates	100	120	7

Synthetic datasets

The SD1 dataset contains 60,000 associations between variable pairs X and Y. The effect chance was set to 1%. The relationships between the 600 non-independent variable pairs were randomly chosen among 6 different types of functional associations, namely cubic, exponential (2^X), line, parabola, sigmoid, and spike (see Table S3 in [1]). The noise term η is a random variable with uniform distribution in the range of $f(X)$ multiplied by an intensity factor k_η. Different values of k_η were chosen randomly among 18,000 values obtained joining the following three sequences: the first ranging from 0.05 to 1 (with steps of 0.0001), the second ranging from 1 to 2 (with steps of 0.0002), and the third ranging from 2 to 9 (with steps of 0.002). Using these values, the coefficients of determination (R^2) between Y and the noiseless function $f(X)$ ranges approximately from 0 to 1. The remaining 99% (59,400) associations were defined with X and Y randomly generated from a uniform distribution between 0 and 1. To characterize the effect of the sample size, we created 20 replicates of SD1 for an increasing number of samples ($n \in \{25, 50, 100, 250, 1{,}000\}$), for a total of 100 datasets. Considering that the fraction of true positive associations was known, this design of experiment allowed us to quantify the statistical power and the performances in terms of FDR of the proposed pipeline. The results for 2×10^5 permutations are summarized in Figure 2 and in the Additional File 1, Table A1. The dependence of the power and of the number of false positives (FP) from the number of samples are shown in Figure 2a and 2b. The power increases with the number of samples reaching 75% for a sample size of 100. As expected, considering that we used the Storey's q value as a strategy to control the FDR, also the number of false positive grows for increasing sample size (Figure 2b) to keep the false discovery rate constant (0.05 in this case). Figure 2c shows the observed FDR, which is almost equal to the expected value of 0.05 for all samples sizes. In Figure 2d we show the values of MIC$_e$ as a function of the coefficient of determination (R^2) between Y and the noiseless function $f(X)$ for the associations that pass the significance filter (i.e. associations with q values <0.05). As expected, MIC$_e$ and R^2 were always linearly correlated, especially for the larger sample sizes [5] (Figure 2d, upper panel). Moreover, we found that, for small sample sizes, only relationships with relatively high values of R^2 passed the significance filter. This effect decreases with increasing number of samples, showing that the pipeline is able to identify relationships with more noise, provided that a sufficient number of experimental points is available. This effect is clearly visible in Figure 2e, where we show the statistical power as a function of the strength of the relationships for different sample sizes. While on less noisy associations (having R^2 close to 1) the pipeline shows high power also for smaller sample sizes, a high number

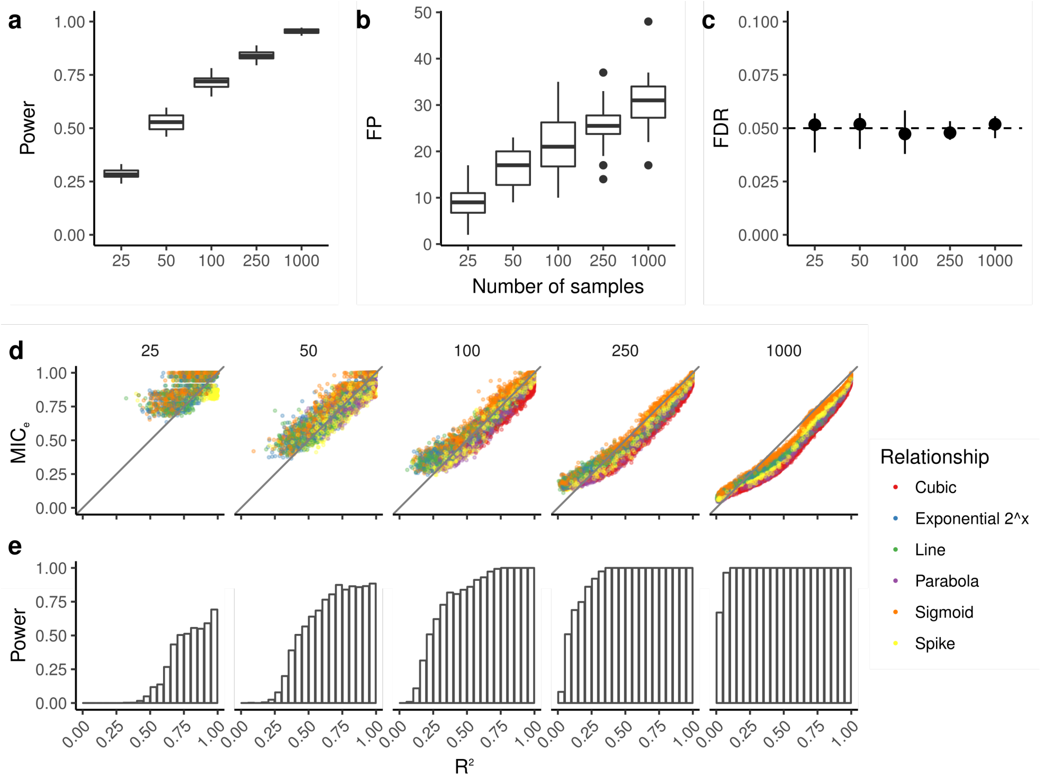

Figure 2. Analysis on SD1 dataset at the 0.05 significance level. (a) Statistical power, (b) number of false positives (FP), and (c) false discovery rate (FDR) at varying number of samples n. Each range represents the results of the 20 replicates. (d) MIC_e values and (e) statistical power at different levels of R^2, for increasing number of samples (from 25 to 1,000, plots from left to right). Only significant relationships, i.e. relationships with $q < 0.05$, are shown.

of samples is needed to attain high power for very noisy relationships (having R^2 close to 0). Upon closer inspection, the panel d in Figure 2 also shows that the power depends on the form of the association. For instance, red points (corresponding to cubic functional forms) are hardly visible for sample sizes smaller than 100, while sigmoidal, linear and exponential relationships can be identified for all sample sizes, albeit with a power that depends on the amount of noise. This finding can be easily interpreted considering that more complex relationships (e.g. polynomials of higher order) are defined by a higher number of parameters that makes them more difficult to distinguish from random associations if the number of points is limited. A more clear representation of this phenomenon is included in Additional File 2 (Figure A1). Moreover, the downward bias in terms of equitability, especially for the more complex relationships (Figure 2d and A1) is a result of the core approximation algorithm EQUICHARCLUMP, which speeds up the computation of MIC_e [18, 5]. The EQUICHARCLUMP parameter c controls the coarseness of the discretization in the grid search phase and by default it is set to 5, providing good performance in most settings [10].

As anticipated, SD1 was also used to investigate the dependence of the performances of MICtools on the number of independent permutations used to estimate the empirical null distribution. Figure A2 and A3 (Additional File 2) shows the FDR and the power as a function of the number of samples and of the number of permutations. The plots indicate that for all the combinations of the two parameters the measured FDR was consistent with the expected value 0.05 (Additional File 2, Figure A2 and Additional File 1, Table A2) and that the true value is always included in the shaded interquartile area. As expected, the variability is stronger for the smaller dataset (25 samples), but also with such a small number of samples above 2×10^5 permutations the median measured FDR stabilizes around 0.05. Figure A3a in the Additional File 2 shows the expected increase of power with the number of samples, from 0.25 to almost 1. The median values does not show a strong dependence on the number of permutations. Figure A3b indicates that below 100 samples at least 2×10^5 permutations are needed to obtain stable values of power, and that its variability is anyway larger for small sample sets. In MICtools, the default value of the number of permutations is set to 2×10^5 and the parameter can be optimized by the user on the bases of the characteristics of the dataset under analysis.

The dataset SD2 was generated to characterize how the effect chance, i.e. the fraction of non-random associations, affected the performances of MICtools. Similarly to dataset SD1, SD2 contains a subset of variable pairs X and Y related by associations of the form $Y = f(X) + \eta$, where η was defined as in SD1. The number of samples was fixed to $n = 100$ and the total number of associations was 60,000. For each effect chance value (1%, 2%, 5%, 10%, 20% and 50%) we generated 20 independent datasets, for a total of 120. The power, number of False Positives (FP) and FDR as a function of the effect chance are shown in Figure 3, panels a, b and c, respectively (see also Additional File 1, Table A3). In Figure 3a, we can observe that the statistical power grows with the effect chance, while the actual FDR remains constant. In fact, an increase of effect chance corresponds to a decrease of the fraction of relationships for which the null is true, π_0 (effect chance = $1 - \pi_0$). Consequently, an increase of the p-value threshold and therefore a growth of power is expected in order to maintain the FDR cutoff constant [7, 14].

The Madelon classification dataset

The analysis of SD1 and SD2 datasets demonstrates that MICtools is able to identify the relationships described by analytic functions with additive noise. However, more general forms of

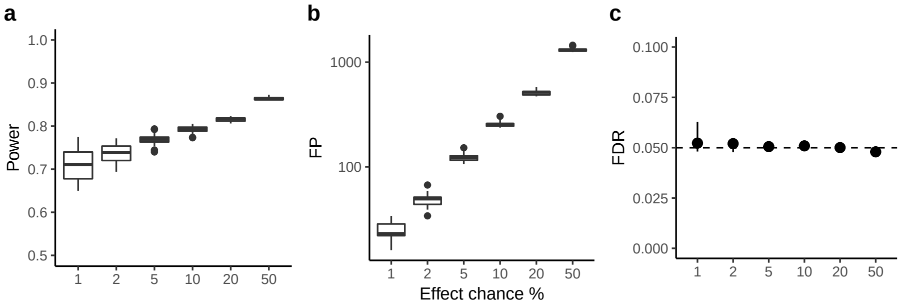

Figure 3. Analysis on SD2 dataset at the 0.05 significance level. (a) Statistical power, (b) number of False positive (FP), and (c) False discovery rate (FDR) for increasing effect chance. Each range represents 20 replicated datasets.

non-random associations are possible. Consider, for instance, the presence of clusters that might indicate the presence of subpopulations. To test the ability of MICtools to identify this type of associations, we created 7 datasets with an increasing number of samples $n \in \{50, 250, 500, 1{,}000, 2{,}500, 5{,}000\}$ with a structure similar to the Madelon binary classification dataset [16, 15] (http://archive.ics.uci.edu/ml/machine-learning-databases/madelon/Dataset.pdf) using the `datasets.make_classification()` function available in the scikit-learn library [19]. Each dataset contains 4 clusters (two for each class), placed on the vertices of a five dimensional four-sided hypercube. Each cluster was composed by normally distributed points ($\sigma = 1$). The five dimensions defining the hypercube constitutes the 5 "informative" features. Other 15 "redundant" features were generated as random linear combinations of the informative features and added to the dataset. Finally, 180 random variables without predictive power were added, for a total of 200. In this type of setting, the number of associations to be tested was 19900 = $(200 \times 199)/2$. Among them, 190 are "real" (the relationships between the variables belonging to the "informative" and "redundant"). Figure 4a summarizes the results of the analysis. Panel (a) shows the association called significant (q-value cutoff set to 0.05) on a Hive plot [20] as a functions of the number of samples. Each branch of the Hives represents a type of variable (informative: 5 variables; redundant: 15; random: 180), the blue lines identify true positives (associations between non-independent variables correctly identified), while false positives (incorrectly identified associations between independent variables) are marked in red. This representation clearly shows that, as expected, the number of true positives increases with the number of samples. A more quantitative representation of the effect of the number of samples on the number of false negatives (non-independent associations incorrectly rejected) is shown in panel (b). Again, an increase in the number of samples is beneficial, because it reduces the number of false negatives. The last panel (c) of Figure 4 shows the effect of n on the FDR, which is always approximately constant and very close to the theoretical value of 0.05.

On the bases of these results, we conclude that also with a relatively low number of samples MICtools is able to identify in an efficient way non functional associations typical of cluster structures. It is interesting to note that the associations among the informative variables started to be recovered when at least 250 samples were considered, while the associations between informative/redundant and redundant/redundant variables were significant also for lower number of samples (50). This apparently odd behavior is due to the different nature of the association among the variables. Binary associations among informative variables are indeed characterized by the presence of clusters, while redundant associations are constructed by linear combinations. In accordance to the results discussed for SD1, the statistical power of the procedure depends on the type of association and with a lower number of samples the results are biased towards less complex association patterns.

Identifying ecological niches: the Tara dataset

The Tara Oceans project is a large multinational effort for the study of plankton at a global scale [17]. Within the project, a large study of the microbiota of water samples from the oceans characterized using metagenomic techniques has been recently made available. To illustrate the added value of using MICtools to analyze such large datasets, we downloaded the annotated 16S $_{\mathrm{mi}}$tags [21] OTU count table of 139 water samples from http://ocean-microbiome.embl.de/companion.html, together with the accompanying metadata on temperature and chemical composition [22]. MICtools was used to identify the existence of significant relationships between the environmental variables and the taxonomic composition of the microbiota. The genus relative abundances, the environment variables and the samples metadata are available in the Additional File 1, tables A4, A5 and A6 respectively. By using a q-value cutoff of 0.01 we found significant associations between the relative abundances of 279 taxa with water temperature and of 287 taxa with oxygen (Figure 5, panels b and c, respectively). To highlight the novel information provided by MICtools, Spearman's rank correlation coefficients and their associated p values were also calculated as in [23] (the default for the `cor.test()` function in the R environment). By using the Spearman's coefficient alone we could identify a subset of the relations identified by MICtools, namey 194 taxa were associated with temperature and 191 taxa were associated with oxygen concentration, respectively. Conversely, almost all relationships identified with Spearman's correlation were also identified by MICtools. While the Spearman's coefficient based approach identified associations well described by monotonic functions (Figure 5e and 5f), MICtools was able to highlight the presence of more complex relationships between the taxa and the environmental parameters. As an example, we found a sharp increase of the Alcaligenaceae genus at oxygen concentration of 200 μmol kg^{-1} (Figure 5d) and a slow increase of the Sphingomonadaceae genus as a function of the temperature. In both cases, highlighting the samples on the bases of their specific aquatic layer of reference it is possible to see that the complex aggregation patterns identified by MICtools are associated to specific ecological niches. These results show the advantage of the use of the proposed approach as an automatic screening tool in the data exploration phase. The lists of the relationships identified by MICtools and by the Spearman-based procedure are available in the Additional File 1, Tables A7 and A8, respectively.

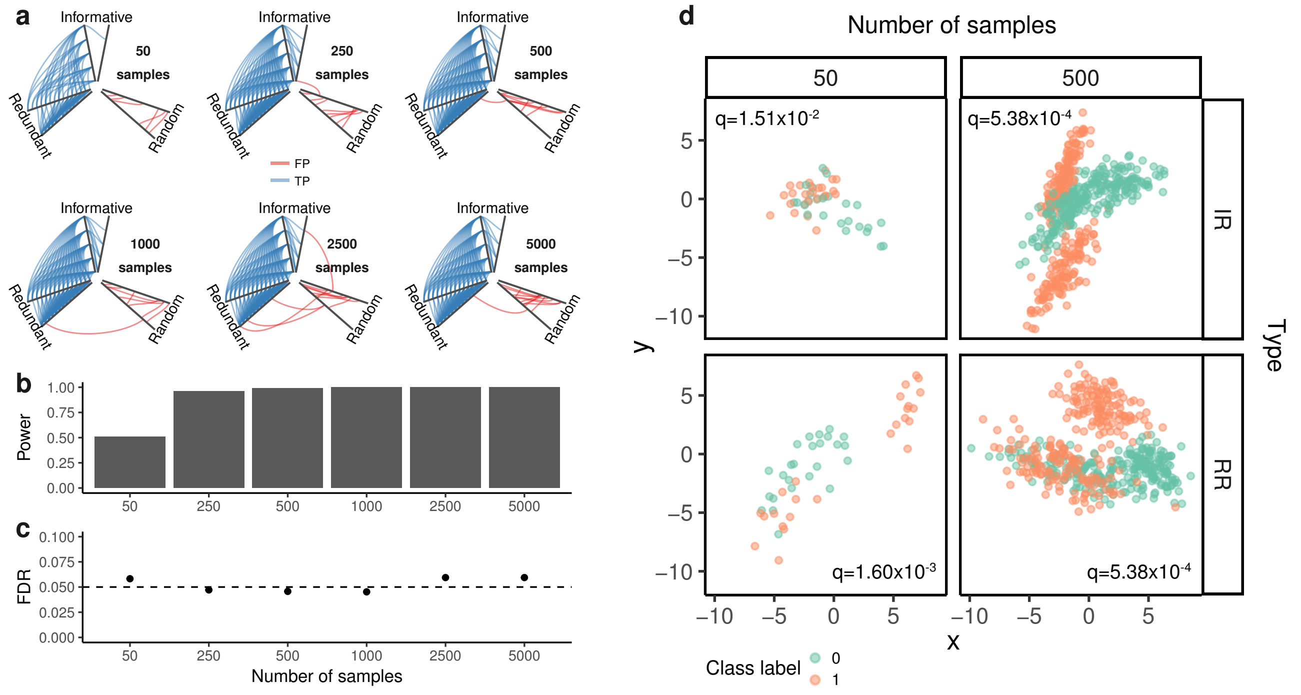

Figure 4. Madelon dataset. (a) Hive plots of the detected association for increasing number of samples. The variables are grouped as "informative" (5), "redundant" (15), and "random" (180). True positives (associations between non-independent variables passing the significance test) are in blue; false positives (associations between independent variable passing the significance test) in red. (b) Power, and (c) false discovery rate (FDR) as a function of the number of samples. (d) Example of significant relationships between informative and redundant (IR) and redundant (RR) variables within the Madelon datasets with 50 and 500 samples.

Implementation details

MICtools is a Python-based open source software (licensed under GPLv3). MICtools requires the minepy [11] (https://minepy.readthedocs.io), Statsmodels [24] and the NumPy, SciPy, pandas and Matplotlib scientific libraries. MICtools can handle different types of experiments:

- given a single dataset X with M variables and n samples, MICtools evaluates the $\frac{M\times(M-1)}{2}$ possible associations;
- given two datasets, X (of size $M \times n$) and Y (of size $K \times n$) MICtools evaluates all the pairwise relationships between the variables of the two datasets (for a total of $M \times K$ associations).
- given two datasets, X (of size $M \times n$) and Y (of size $K \times n$) it evaluates all the row-wise relationships, i.e. only the variables pairs x_i and y_i (for $i = 1, \ldots, \min(M, K)$) will be tested;
- moreover, for each experiment listed above, if the sample classes are provided, the analysis will be performed within each class, independently.

For multiple testing correction MICtools makes available the strategies implemented in Statsmodels and a Python implementation of the Storey's q-value method [7]. The indicative number of relationships tested per second during the empirical null estimation (using the TIC_e) and the strength estimation (MIC_e) for an increasing number of samples are reported in Additional File 2, Figure A3.

MICtools source and the documentation is available at https://github.com/minepy/mictools. The Docker (https://www.docker.com/) image, containing MICtools and the minepy library is available at https://hub.docker.com/r/minepy/mictools/ and installable with the command `docker pull minepy/mictools`.

Availability of source code and requirements

- Project name: MICtools
- Project home page: https://github.com/minepy/mictools
- Research Resource Identification Initiative ID (RRID), SciCrunch.org: SCR_016121
- Operating system(s): Platform independent
- Programming language: Python
- Other requirements: minepy, Statsmodels, NumPy, SciPy, pandas, Matplotlib
- License: GNU GPLv3

Availability of supporting data and materials

The Tara dataset is available at http://ocean-microbiome.embl.de/companion.html.

Declarations

List of abbreviations

FDR: False Discovery Rate; FN: False Negative; FP: False positive; FWER: Family-Wise Error Rate; MIC: Maximal Information Coefficient; SD1: Synthetic dataset 1; SD2: Synthetic dataset 2; TIC: Total Information Coefficient

Ethical Approval (optional)

Not applicable.

Consent for publication

Not applicable.

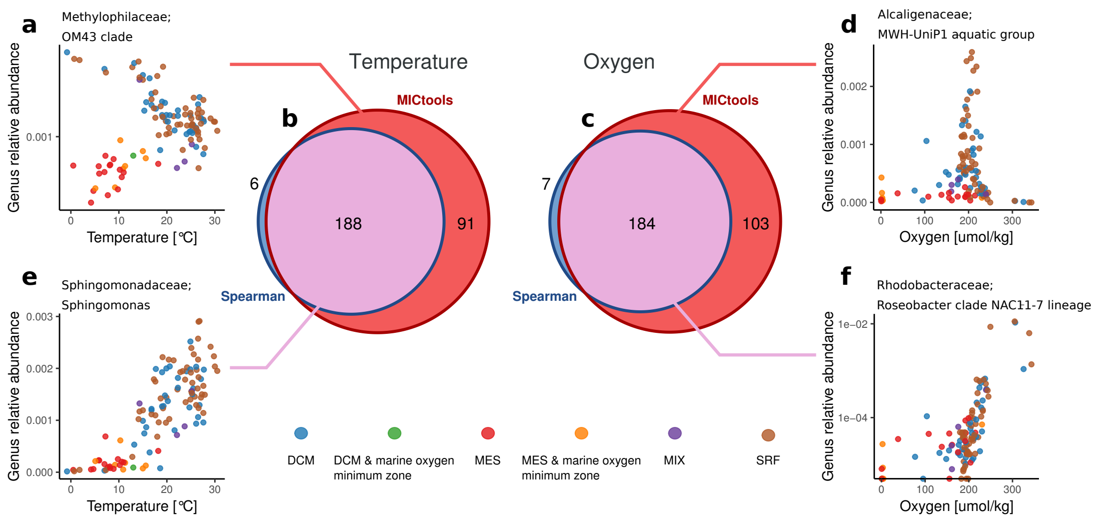

Figure 5. Tara dataset: venn diagrams of the significant relationships between the genus-level relative abundances and two environmental variables, temperature (b) and oxygen (c) identified by MICtools and the Spearman-based procedure ($q < 0.01$). (a, d): the relationships between the OM43 clade and the temperature and between the MWH-UniP1 aquatic group are detected only by MICtools. (e, f): two monotonic relationships identified by both methods. Abbreviations: DCM, deep chlorophyll maximum layer; MES, mesopelagic zone; MIX, subsurface epipelagic mixed layer; SRF, surface water layer.

Competing Interests

The authors declare that they have no competing interests.

Funding

This research was supported by the Autonomous Province of Trento (Accordo di Programma).

Author's Contributions

D.A., S.R., C.D. and P.F. conceived the manuscript. D.A. and P.F. developed the methodology. D.A. wrote the software. DA, SR and C.D analyzed the data. D.A., S.R., C.D. and P.F. wrote the manuscript.

Acknowledgements

Not applicable.

References

1. Reshef DN, Reshef YA, Finucane HK, Grossman SR, McVean G, Turnbaugh PJ, et al. Detecting Novel Associations in Large Data Sets. Science 2011;334(6062):1518–1524.
2. Kinney JB, Atwal GS. Equitability, mutual information, and the maximal information coefficient. Proceedings of the National Academy of Sciences 2014;111(9):3354–3359.
3. Murrell B, Murrell D, Murrell H. R2-equitability is satisfiable. Proceedings of the National Academy of Sciences 2014;111(21):E2160–E2160.
4. Reshef DN, Reshef YA, Mitzenmacher M, Sabeti PC. Cleaning up the record on the maximal information coefficient and equitability. Proceedings of the National Academy of Sciences 2014;111(33):E3362–E3363.
5. Reshef YA, Reshef DN, Finucane HK, Sabeti PC, Mitzenmacher M. Measuring Dependence Powerfully and Equitably. J Mach Learn Res 2016;17(212):1–63.
6. Reshef YA, Reshef DN, Sabeti PC, Mitzenmacher MM. Equitability, interval estimation, and statistical power. arXiv preprint 2015 May;.
7. Storey JD, Tibshirani R. Statistical significance for genomewide studies. Proc Natl Acad Sci U S A 2003 Aug;100(16):9440–9445.
8. Franceschi P, Giordan M, Wehrens R. Multiple comparisons in mass-spectrometry-based -omics technologies. Trends Analyt Chem 2013;50:11–21.
9. Simon N, Tibshirani R. Comment on "Detecting Novel Associations In Large Data Sets" by Reshef Et Al, Science Dec 16, 2011 2014 Jan;.
10. Reshef DN, Reshef YA, Sabeti PC, Mitzenmacher MM. An Empirical Study of Leading Measures of Dependence. arXiv preprint 2015 May;.
11. Albanese D, Filosi M, Visintainer R, Riccadonna S, Jurman G, Furlanello C. minerva and minepy: a C engine for the MINE suite and its R, Python and MATLAB wrappers. Bioinformatics 2012;29(3):407–408.
12. Storey JD. A direct approach to false discovery rates. J R Stat Soc Series B Stat Methodol 2002;64(3):479–498.
13. North BV, Curtis D, Sham PC. A note on the calculation of empirical P values from Monte Carlo procedures. Am J Hum Genet 2002 Aug;71(2):439–441.
14. Krzywinski M, Altman N. Points of significance: Comparing samples—part II. Nat Methods 2014;11(4):355–356.
15. I G, A E. An Introduction to Feature Extraction. In: I G, M N, S G, LA Z, editors. Feature Extraction. Studies in Fuzziness and Soft Computing, vol. 207 Springer; 2006.
16. Guyon I, Gunn S, Nikravesh M, Zadeh LA. Feature Extraction: Foundations and Applications. Springer; 2008.
17. Bork P, Bowler C, de Vargas C, Gorsky G, Karsenti E, Wincker P. Tara Oceans. Tara Oceans studies plankton at planetary scale. Introduction. Science 2015 May;348(6237):873.
18. Reshef D, Reshef Y, Mitzenmacher M, Sabeti P. Equitability analysis of the maximal information coefficient, with comparisons. arXiv preprint arXiv:13016314 2013;.
19. Pedregosa F, Varoquaux G, Gramfort A, Michel V, Thirion B, Grisel O, et al. Scikit-learn: Machine Learning in Python. Journal of Machine Learning Research 2011;12:2825–2830.
20. Krzywinski M, Birol I, Jones SJM, Marra MA. Hive plots–rational approach to visualizing networks. Brief Bioinform 2012 Sep;13(5):627–644.

21. Logares R, Sunagawa S, Salazar G, Cornejo-Castillo FM, Ferrera I, Sarmento H, et al. Metagenomic 16S rDNA Illumina tags are a powerful alternative to amplicon sequencing to explore diversity and structure of microbial communities. Environ Microbiol 2014 Sep;16(9):2659–2671.
22. Sunagawa S, Coelho LP, Chaffron S, Kultima JR, Labadie K, Salazar G, et al. Ocean plankton. Structure and function of the global ocean microbiome. Science 2015 May;348(6237):1261359.
23. Best DJ, Roberts DE. Algorithm AS 89: The Upper Tail Probabilities of Spearman's Rho. Appl Stat 1975;24(3):377.
24. Seabold S, Perktold J. Statsmodels: Econometric and statistical modeling with python; 2010. .

Additional File 1

Click here to access/download
Supplementary Material
Additional File 1(1).xlsx

Additional File 2

Click here to access/download
Supplementary Material
Additional File 2.pdf

Manuscript - Track changes

Click here to access/download
Supplementary Material
main_track_changes.pdf

Click here to access/download
Supplementary Material
Reference PDF.pdf
