# Supplementary material for: A practical tool for maximal information coefficient analysis
Source: Gigascience. 2018 Apr 2;7(4):1–8. doi: 10.1093/gigascience/giy032 (PMC5893960; doi:10.1093/gigascience/giy032)
Supplement: Additional Files [file giy032_supp.zip › Additional File 2.pdf]

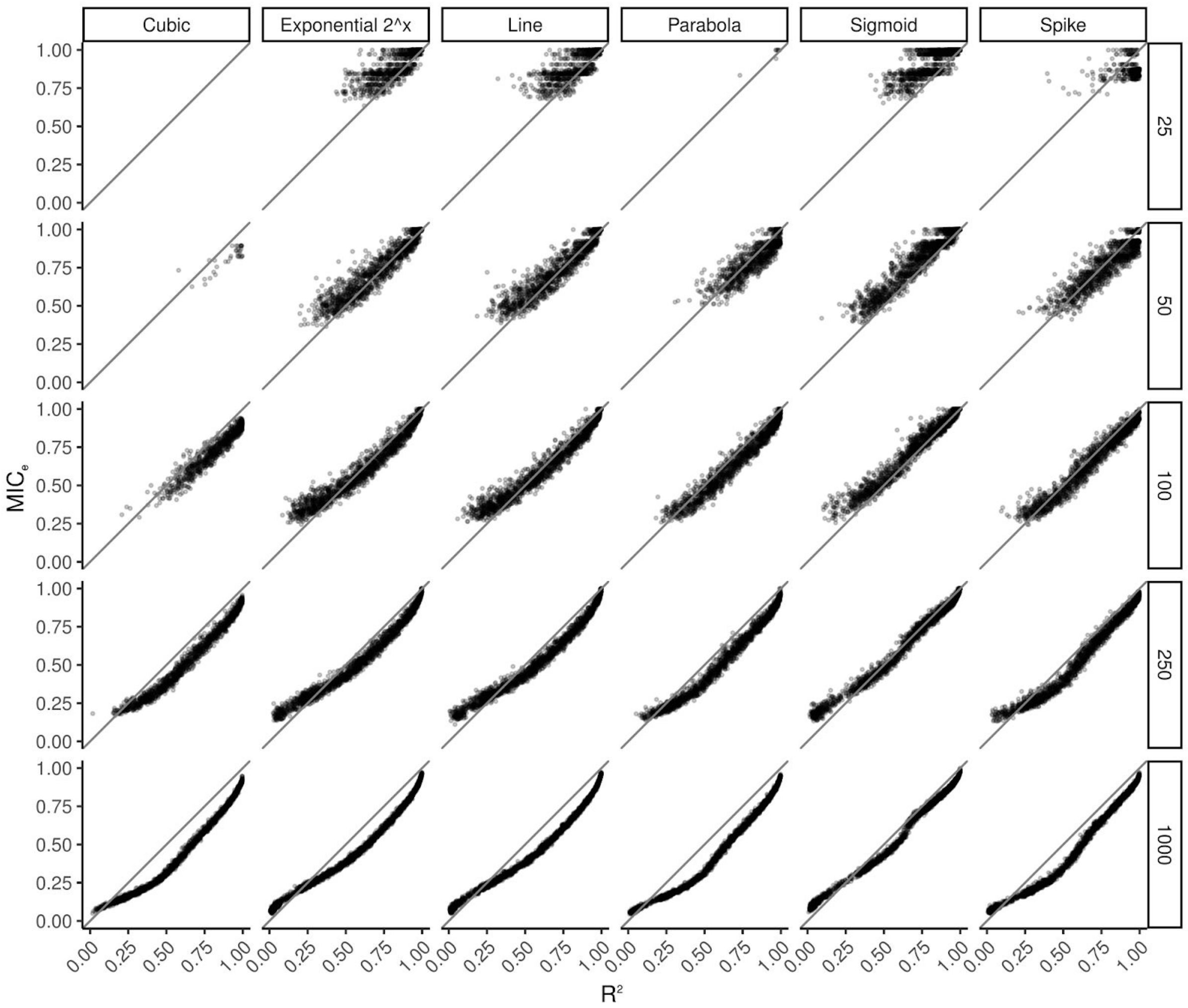

Figure A1: SD1 dataset. Relation between MIC_e and R^2 for different types of functional relationships, for increasing number of samples. As discussed in the main text, R^2 and MICe are always linearly correlated. The relation, however, is more noisy for smaller sample sizes. A decrease in the sample size does not only affect the variability of the linear association, but also depends on the specific functional form. For small sample sizes MICtools identifies preferentially associations with simple forms and high R^2.

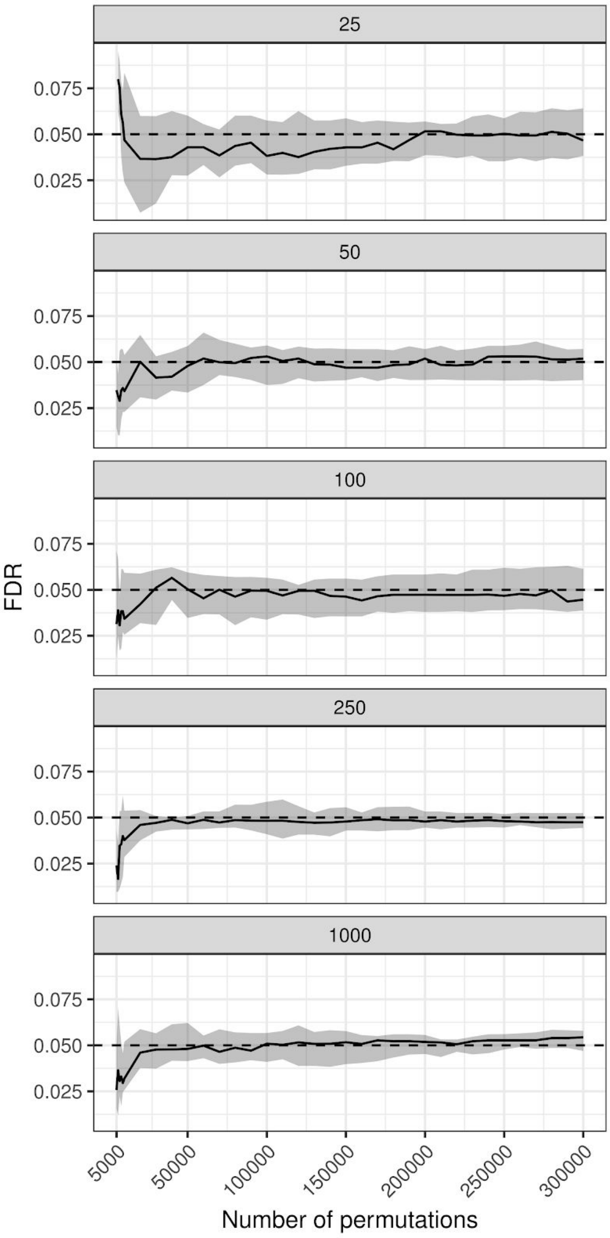

Figure A2. Dataset SD1. False discovery rate (FDR) for increasing sample sizes (from 25 to 1,000) and number of permutations. The solid lines indicate the medians of the 20 independent replicates of the dataset. The lower and upper bounds of the grey ribbons indicate the first and the third quartile, respectively.

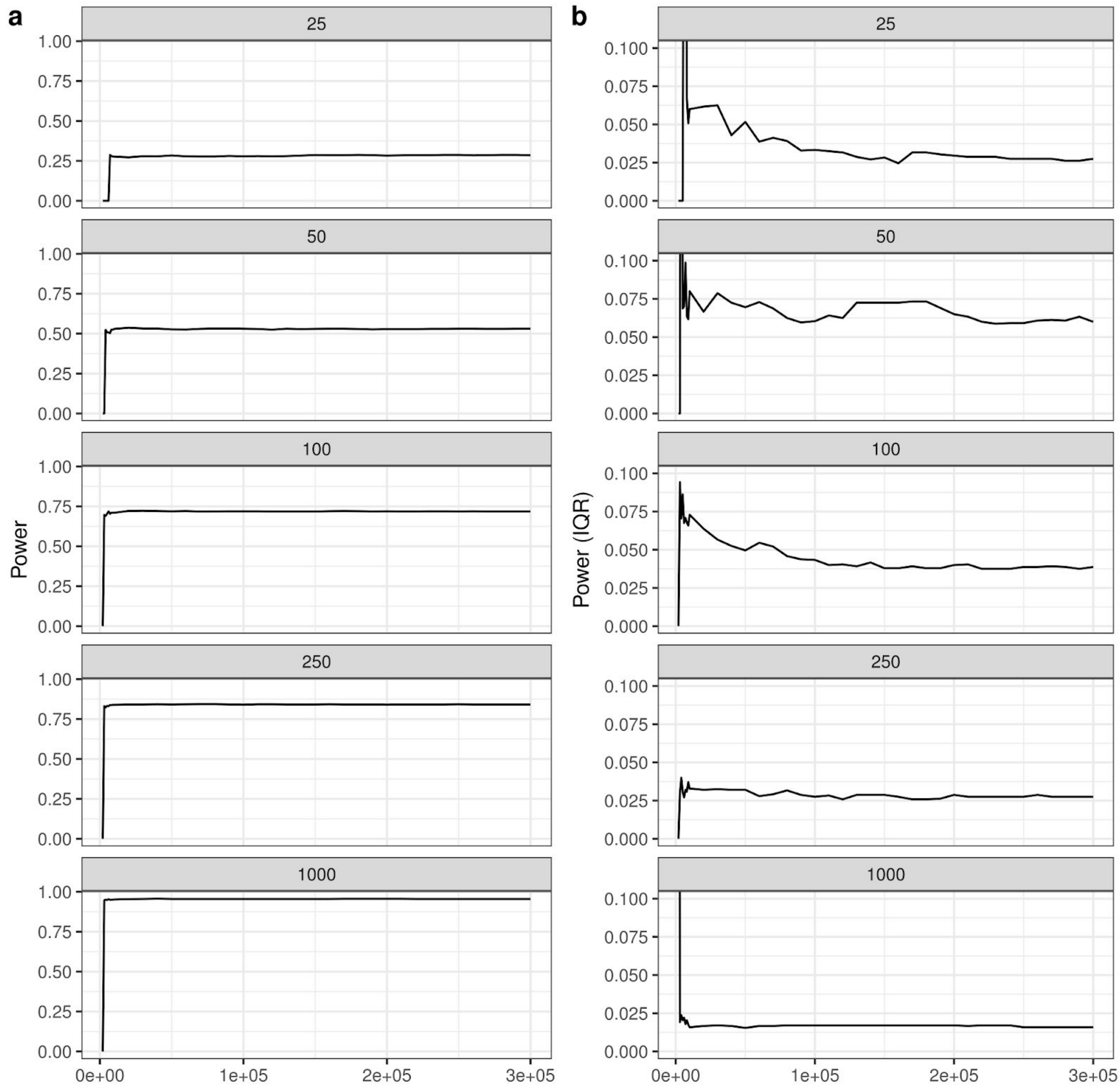

Figure A3. Dataset SD1. Median power (a) and inter-quartile range (IQR) (b) for increasing sample sizes (from 25 to 1,000) and number of permutations (from 1 to 3 x 10^5).

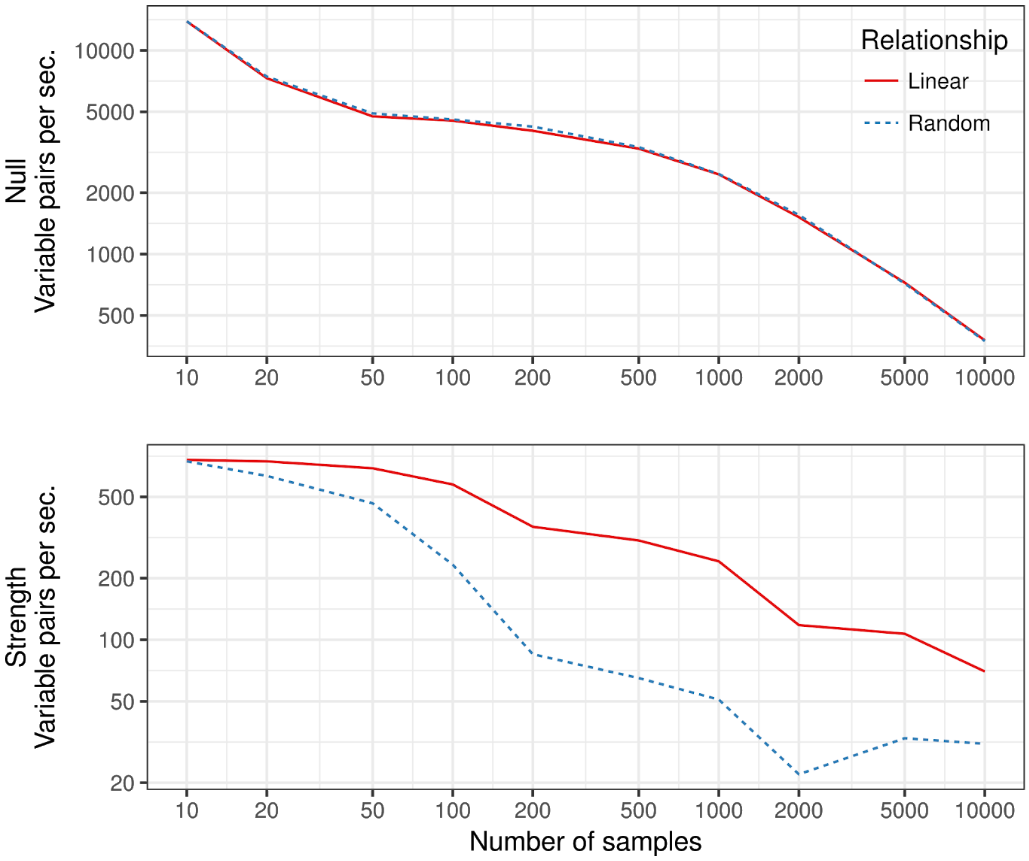

Figure A4. Number of relationships tested per second during the empirical null estimation (`mictools null` command, upper panel) and the strength estimation (`mictools strength` command, lower panel) for an increasing number of samples, from 10 to 10,000. The values reported refers to perfectly linear (in red) and random (in blue) associations. The test was performed on a notebook with an Intel® Core™ i7-7560U CPU @ 2.40GHz × 4 and 16 GB of RAM.
