# Supplementary material for: A practical tool for maximal information coefficient analysis
Source: Gigascience. 2018 Apr 2;7(4):1–8. doi: 10.1093/gigascience/giy032 (PMC5893960; doi:10.1093/gigascience/giy032)
Supplement: Response_to_Reviewer_Comments_Original_Submission.pdf [file giy032_response_to_reviewer_comments_original_submission.pdf]

Author's Response To Reviewer Comments

00000000-0000-(QRw YYvjFalEps

E2A9317A hPTrimD/Kw sE0M

Close

Dear Editor,
on behalf of all authors, I’m pleased to submit the revised manuscript ”A practical tool for Maximal Information Coefficient analysis” by Albanese et al., to be considered for publication in Gigascience as Technical Note.

We are grateful to the referees for their time and efforts: their comments has been extremely useful in improving the overall quality of the manuscript and we incorporated all their suggestion in the revised text. Hereafter, the answers to the referees comments are highlighted by the string ">>>".

The authors declare no conflict of interest and have approved the manuscript for submission. I hereby confirm that the present work has not been published and submitted for publication elsewhere.

Editor’s comments

Please register any new software application in the SciCrunch.org database to receive a RRID (Research Resource Identification Initiative ID) number, and include this in your manuscript. This will facilitate tracking, reproducibility and re-use of your tool.

>>> The software tool has been registered in SciCrunch as SCR_016121. This information has been included in the manuscript.

A third referee declined to review as they had very strong views against maximal information coefficient (MIC), feeling it was essentially a messed-up estimator of mutual information, and that any research based on it was flawed. Consulting with the other referees they disagreed with this, but it does seem there are strong views on this technique. It is probably not possible to satisfy both sides of this debate, but if there are any ways to acknowledge this in the discussion that could potentially be useful.

>>> We are aware the the introduction of MIC has been triggering a lively discussion in the scientific community. In order to acknowledge that we added a short sentence in the introduction.

Reviewer #1

In this paper the authors describe and analyse a series of tools to find complex associations in large omics data sets. At the core of these tools lies the measure of association Maximal Information Coefficient (MIC) which recently received a lot of interest in data mining community. Other than presenting the first publicly available implementation of MIC to date, the authors make available the code for a complete pipeline to identify statistically significant associations between the features in a data set. This involves:
- Computing the Total Information Coefficient (TIC) for each pair of features
- Computing their p-value using a permutation test with Monte Carlo simulations
- Select the significant pairs using statistical correction for multiple hypotheses
- Rank the statistically significant associations according to MIC

Moreover, the authors analyse the results of their pipeline on synthetic and real data sets. I commend the authors for providing the community with a well-tested implementation of MIC (and its more recent version MIC_e) in various programming languages including C, Matlab, and Python. I also really appreciate publishing a full pipeline to identify associations between features written in Python, which is probably the most popular language in the data science community. Moreover, the paper is well written and the analyses about the effectivity of these tools are convincing. The paper should be accepted for publication in the GigaScience journal. There has been so much discussion about the merit of MIC in the past years since its publication in 2011. I am honestly impressed by MIC's authors efforts to shed light on the theoretical and empirical properties of MIC. Their effort recently found venue in prestigious

journals such as the Proceedings of the National Academics of Science (PNAS) in 2014, the Journal of Machine Learning Research (JMLR) in 2016, and the Annals Of Applied Statistics (AOAS) in 2017. The main criticism about MIC has been its similarity to one of the many estimators of mutual information. Even though MIC exploits mutual information, MIC has been shown to not be the same as estimating mutual information [Measuring dependence powerfully and equitably by Reshef et al. in JMLR 2016]. Nonetheless, what strikes me the most is that: in many empirical studies no estimator of mutual information has the same performance of MIC in terms of equitability. Being equitability a very intuitive property, I do understand why researchers and data mining practitioners value MIC. I have only one concern about the methodology of screening associations with TIC and ranking only the selected ones with MIC. Possibly if we are interested just in equitability, MIC should be the only association measure to be employed in the analysis. However, given than TIC shows to have more power the MIC [An Empirical Study of the Maximal and Total Information Coefficients and Leading Measures of Dependence by Reshef et al. in AOAS 2017], I guess that the associations that MIC would deem as significant would be a subset of the significant associations for TIC.

>>> We thank the reviewer for his comment and exactly for the reasons he mention we decided to rely on a two step procedure to look for an optimal trade-off between speed/power and equitability

Minor comments:
It would be great to describe the Storey's method to control the FDR in the paper to make it self-contained; It would be also great to briefly describe the procedure to control the FWER;

>>>The section describing how multiple testing correction was performed has been extended including:
>>> - a definition of FWER and FDR
>>> - an explicit description of the key idea behind Storey's q-value

A table describing the difference between the data sets SD1 and SD2 would be informative. Possibly a line describing the Madelon semi-synthetic data sets would be useful too

>>> A description of the characteristics of the two synthetic dataset gas been included in the main text as Table 2

The authors discuss a great insight on MIC when they say that: "associations between informative/redundant and redundant/redundant variables were significant also for a lower number of samples". It would be nice to have a visual example about these type of associations;

>>> Figure 4 was modified to include four visual examples of the associations found in the Madelon dataset. In particular two examples of IR and RR associations for 50 and 500 samples are included in the panel (d) of the revised figure.

Figure 4 b. I guess discussing a decreasing FN is the same as discussing increasing power. Changing the FN plot in a power plot would make the paper more coherent: eg as in Figure 2 a;

>>> The figure was corrected following the Referee's suggestion

"coniugate" in the abstract -> conjugate. Maybe better to reformulate this sentence as it is not very clear;

>>> The corresponding sentence has been rephrased also according to the comments of Reviewer #2

Reviewer #2

This manuscript introduces an open-source implementation of two measures of dependence, MICe and TICe, which together provide a combination of both statistical power and equitability for identifying associations in large data sets. The implementation provided by the authors is a valuable contribution to the community that allows for the easy computation of these measures of dependence, and I'd recommend its acceptance after the authors make the minor edits listed below.

Minor Comments
A few minor comments that the authors should be made aware of (but that I didn't want to be public given how minor they are):

There are a few small type-o's to correct (e.g. coniugate on Pg. 1, line 31; expenses on pg. 2, line 15).

>>> Corrected

I would suggest the authors soften the language around the fact that "an implementation of these two measures and of a statistical procedure to test the significance of each association is still missing." The authors who developed MICe and TICe are simply waiting to post their implementation of MICe and TICe at www.exploredata.net along with the official publication of the most recent paper analyzing these measures in the Annals of Applied Statistics (https://www.e-publications.org/ims/submission/AOAS/user/submissionFile/29563?confirm=583655c8). That said, the implementation in this manuscript submitted to GigaScience is still a valuable contribution as it is open-source (the implementation AOAS will post is not) and provides a more comprehensive procedure to test for significance.

>>> The text has been corrected following the referee's advice

On Pg. 1, line 31, "which coniugate computational efficiency with good bias/variance properties", isn't quite accurate. I'd change this to "which combine computational efficiency with superior bias/variance properties".

>>> Changed

On Pg. 2, line 5, "has been shown to satisfy the equitability requirement" should be changed to "has been shown to have good equitability" to reflect the fact that equitability is not a binary property, but a continuous one that a measure of dependence can have more or less of.

>>> Changed

On Pg. 2, line 6 - MIC doesn't actually suffer from lack of power, and this fact has been corrected in the literature, so I would recommend using softer language. It was shown in ref. 12 that was cited by the authors that the original perceived bad power of MIC was due to incorrect parameter settings by those who drew that conclusion. When used with appropriate parameters for independence testing, MIC has decent, but not state-of-the-art, power. What is accurate, however, is that MICe and TICe *improved* upon the power of MIC, and that TICe has state-of-the-art power.

>>> "suffers of lack of power" has been changed with "does not have state-of-the-art power"

On Pg. 2, second column, line 23, regarding the sentence beginning with "With regards to the number of permutations..." (and elsewhere): the number of permutations necessary to perform for any given analysis scales with the number of tests one must correct for (i.e. the number of variable pairs for which a measure of dependence was computed), as the FDR accuracy is inversely proportional to the number of permutations used to compute it, so I'd be careful about saying that a specific number is generally enough for data of any dimensionality.

>>> The text of the paper was changed according to the referee's comments. In particular we highlighted the fact that the the number of permutations is a parameter that can be adjusted by the user on the bases of the dataset characteristics.

Close
