# Supplementary material for: A practical tool for maximal information coefficient analysis
Source: Gigascience. 2018 Apr 2;7(4):1–8. doi: 10.1093/gigascience/giy032 (PMC5893960; doi:10.1093/gigascience/giy032)
Supplement: Response_to_Reviewer_Comments_Revision_1.pdf [file giy032_response_to_reviewer_comments_revision_1.pdf]

Author's Response To Reviewer Comments

00000000-0000-(	2Qb+maKCZMJz(
E2A9317A	n4zmM09W/sBD1

Close

>The main issue is to incorportate the data DOI into the "Availability of supporting data section", mentioning that code snapshots and tabular test data is available in the GigaScience GigaDB repository. You then need to cite the dataset (probably as reference 25) and include the following citation in the references:

>Albanese, D; Riccadonna, S; Donati, C; Franceschi, P (2018): Supporting data for "A practical tool for Maximal Information Coefficient analysis" GigaScience Database. http://dx.doi.org/10.5524/100427

We added the required reference and changed the text accordingly.

Close
