# Supplementary material for: A practical tool for maximal information coefficient analysis
Source: Gigascience. 2018 Apr 2;7(4):1–8. doi: 10.1093/gigascience/giy032 (PMC5893960; doi:10.1093/gigascience/giy032)
Supplement: Reviewer_1_Report_(Original_Submission) -- Simone Romano — 2/3/2018 Reviewed [file giy032_reviewer_1_report_(original_submission).pdf]

Reviewer Report

Title: A practical tool for Maximal Information Coefficient analysis

Version: Original Submission **Date:** 2/3/2018

Reviewer name: Simone Romano

Reviewer Comments to Author:

In this paper the authors describe and analyse a series of tools to find complex associations in large omics data sets. At the core of these tools lies the measure of association Maximal Information Coefficient (MIC) which recently received a lot of interest in data mining community. Other than presenting the first publicly available implementation of MIC to date, the authors make available the code for a complete pipeline to identify statistically significant associations between the features in a data set. This involves:- Computing the Total Information Coefficient (TIC) for each pair of features- Computing their p-value using a permutation test with Monte Carlo simulations- Select the significant pairs using statistical correction for multiple hypotheses- Rank the statistically significant associations according to MICMoreover, the authors analyse the results of their pipeline on synthetic and real data sets.I commend the authors for providing the community with a well-tested implementation of MIC (and its more recent version MIC_e) in various programming languages including C, Matlab, and Python. I also really appreciate publishing a full pipeline to identify associations between features written in Python, which is probably the most popular language in the data science community. Moreover, the paper is well written and the analyses about the effectivity of these tools are convincing. The paper should be accepted for publication in the GigaScience journal.There has been so much discussion about the merit of MIC in the past years since its publication in 2011. I am honestly impressed by MIC's authors efforts to shed light on the theoretical and empirical properties of MIC. Their effort recently found venue in prestigious journals such as the Proceedings of the National Academics of Science (PNAS) in 2014, the Journal of Machine Learning Research (JMLR) in 2016, and the Annals Of Applied Statistics (AOAS) in 2017. The main criticism about MIC has been its similarity to one of the many estimators of mutual information. Even though MIC exploits mutual information, MIC has been shown to not be the same as estimating mutual information [Measuring dependence powerfully and equitably by Reshef et al. in JMLR 2016]. Nonetheless, what strikes me the most is that: in many empirical studies no estimator of mutual information has the same performance of MIC in terms of equitability. Being equitability a very intuitive property, I do understand why researchers and data mining practitioners value MIC.I have only one concern about the methodology of screening associations with TIC and ranking only the selected ones with MIC. Possibly if we are interested just in equitability, MIC should be the only association measure to be employed in the analysis. However, given than TIC shows to have more power the MIC [An Empirical Study of the Maximal and Total Information Coefficients and Leading Measures of Dependence by Reshef et al. in AOAS 2017], I guess that the associations that MIC would deem as significant would be a subset of the significant associations for TIC.Minor comments:- It would be great to describe the Storey's method to control the FDR in the paper to make it self-contained; It would be also great to briefly describe the procedure to control the FWER; - A table describing the difference between the data sets SD1 and SD2 would be informative. Possibly a line describing the Madelon semi-synthetic data sets would be useful too;- The authors discuss a great insight on MIC when they say that: "associations between informative/redundant and redundant/redundant variables were significant also for a lower number of samples". It would be nice to have a visual example about these type of associations;- Figure 4 b. I guess discussing a decreasing FN is the same as discussing increasing power. Changing the FN plot in a power plot would make the paper more coherent: eg as in Figure 2 a;- "coniugate" in the abstract -> conjugate. Maybe better to reformulate this sentence as it is not very clear;Simone Romano

Level of Interest

Please indicate how interesting you found the manuscript: An exceptional article

Quality of Written English

Please indicate the quality of language in the manuscript: Acceptable

Declaration of Competing Interests

Please complete a declaration of competing interests, considering the following questions:

- Have you in the past five years received reimbursements, fees, funding, or salary from an organisation that may in any way gain or lose financially from the publication of this manuscript, either now or in the future?
- Do you hold any stocks or shares in an organisation that may in any way gain or lose financially from the publication of this manuscript, either now or in the future?
- Do you hold or are you currently applying for any patents relating to the content of the manuscript?
- Have you received reimbursements, fees, funding, or salary from an organization that holds or has applied for patents relating to the content of the manuscript?
- Do you have any other financial competing interests?
- Do you have any non-financial competing interests in relation to this paper?

If you can answer no to all of the above, write 'I declare that I have no competing interests' below. If your reply is yes to any, please give details below.

I declare that I have no competing interests

I agree to the open peer review policy of the journal. I understand that my name will be included on my report to the authors and, if the manuscript is accepted for publication, my named report including any attachments I upload will be posted on the website along with the authors' responses. I agree for my report to be made available under an Open Access Creative Commons CC-BY license (http://creativecommons.org/licenses/by/4.0/). I understand that any comments which I do not wish to be included in my named report can be included as confidential comments to the editors, which will not be published.

I agree to the open peer review policy of the journal

To further support our reviewers, we have joined with Publons, where you can gain additional credit to further highlight your hard work (see: https://publons.com/journal/530/gigascience). On publication of this paper, your review will be automatically added to Publons, you can then choose whether or not to claim your Publons credit. I understand this statement.

Yes
