# Supplementary material for: A practical tool for maximal information coefficient analysis
Source: Gigascience. 2018 Apr 2;7(4):1–8. doi: 10.1093/gigascience/giy032 (PMC5893960; doi:10.1093/gigascience/giy032)
Supplement: Reviewer_2_Report_(Original_Submission) -- David Reshef — 3/6/2018 Reviewed [file giy032_reviewer_2_report_(original_submission).pdf]

Reviewer Report

Title: A practical tool for Maximal Information Coefficient analysis

Version: Original Submission **Date:** 3/6/2018

Reviewer name: David Reshef

Reviewer Comments to Author:

General comments---This manuscript introduces an open-source implementation of two measures of dependence, MICe and TICe, which together provide a combination of both statistical power and equitability for identifying associations in large data sets. The implementation provided by the authors is a valuable contribution to the community that allows for the easy computation of these measures of dependence, and I'd recommend its acceptance after the authors make the minor edits listed below.Minor Comments---A few minor comments that the authors should be made aware of (but that I didn't want to be public given how minor they are):1) There are a few small type-o's to correct (e.g. coniugate on Pg. 1, line 31; expenses on pg. 2, line 15).2) I would suggest the authors soften the language around the fact that "an implementation of these two measures and of a statistical procedure to test the significance of each association is still missing." The authors who developed MICe and TICe are simply waiting to post their implementation of MICe and TICe at www.exploredata.net along with the official publication of the most recent paper analyzing these measures in the Annals of Applied Statistics (https://www.e-publications.org/ims/submission/AOAS/user/submissionFile/29563?confirm=583655c8). That said, the implementation in this manuscript submitted to GigaScience is still a valuable contribution as it is open-source (the implementation AOAS will post is not) and provides a more comprehensive procedure to test for significance.3) On Pg. 1, line 31, "which coniugate computational efficiency with good bias/variance properties", isn't quite accurate. I'd change this to "which combine computational efficiency with superior bias/variance properties".4) On Pg. 2, line 5, "has been shown to satisfy the equitability requirement" should be changed to "has been shown to have good equitability" to reflect the fact that equitability is not a binary property, but a continuous one that a measure of dependence can have more or less of.5) On Pg. 2, line 6 - MIC doesn't actually suffer from lack of power, and this fact has been corrected in the literature, so I would recommend using softer language. It was shown in ref. 12 that was cited by the authors that the original perceived bad power of MIC was due to incorrect parameter settings by those who drew that conclusion. When used with appropriate parameters for independence testing, MIC has decent, but not state-of-the-art, power. What is accurate, however, is that MICe and TICe *improved* upon the power of MIC, and that TICe has state-of-the-art power.6) On Pg. 2, second column, line 23, regarding the sentence beginning with "With regards to the number of permutations..." (and elsewhere): the number of permutations necessary to perform for any given analysis scales with the number of tests one must correct for (i.e. the number of variable pairs for which a measure of dependence was computed), as the FDR accuracy is inversely proportional to the number of permutations used to compute it, so I'd be careful about saying that a specific number is generally enough for data of any dimensionality.

Level of Interest

Please indicate how interesting you found the manuscript: An article of importance in its field

Quality of Written English

Please indicate the quality of language in the manuscript: Acceptable

Declaration of Competing Interests

Please complete a declaration of competing interests, considering the following questions:

- Have you in the past five years received reimbursements, fees, funding, or salary from an organisation that may in any way gain or lose financially from the publication of this manuscript, either now or in the future?
- Do you hold any stocks or shares in an organisation that may in any way gain or lose financially from the publication of this manuscript, either now or in the future?
- Do you hold or are you currently applying for any patents relating to the content of the manuscript?
- Have you received reimbursements, fees, funding, or salary from an organization that holds or has applied for patents relating to the content of the manuscript?
- Do you have any other financial competing interests?
- Do you have any non-financial competing interests in relation to this paper?

If you can answer no to all of the above, write 'I declare that I have no competing interests' below. If your reply is yes to any, please give details below.

I co-developed the methodology (MICe and TICe) that this manuscript implements.

I agree to the open peer review policy of the journal. I understand that my name will be included on my report to the authors and, if the manuscript is accepted for publication, my named report including any attachments I upload will be posted on the website along with the authors' responses. I agree for my report to be made available under an Open Access Creative Commons CC-BY license (http://creativecommons.org/licenses/by/4.0/). I understand that any comments which I do not wish to be included in my named report can be included as confidential comments to the editors, which will not be published.

I agree to the open peer review policy of the journal

To further support our reviewers, we have joined with Publons, where you can gain additional credit to further highlight your hard work (see: https://publons.com/journal/530/gigascience). On publication of this paper, your review will be automatically added to Publons, you can then choose whether or not to claim your Publons credit. I understand this statement.

Yes
